# Characterization of the Interrenal Gland and Sexual Traits Development in *cyp17a2*-Deficient Zebrafish

**DOI:** 10.3389/fendo.2022.910639

**Published:** 2022-06-06

**Authors:** Shengchi Shi, Tingting Shu, Xi Li, Qiyong Lou, Xia Jin, Jiangyan He, Zhan Yin, Gang Zhai

**Affiliations:** ^1^ State key Laboratory of Freshwater Ecology and Biotechnology, Institute of Hydrobiology, Chinese Academy of Sciences, Wuhan, China; ^2^ College of Advanced Agricultural Sciences, University of Chinese Academy of Sciences, Beijing, China; ^3^ Chinese Sturgeon Research Institute, China Three Gorges Corporation, Yichang, China; ^4^ Center of Clinical Research, The Affiliated Kangning Hospital of Wenzhou Medical University, Wenzhou, China; ^5^ The Innovative Academy of Seed Design, Chinese Academy of Sciences, Wuhan, China

**Keywords:** zebrafish, *cyp17a2*, cortisol, oocyte maturation, sexual trait

## Abstract

Unlike the Cytochrome P450, family 17, subfamily A, member 1 (Cyp17a1), which possesses both 17α-hydroxylase and 17,20-lyase activities involved in the steroidogenic pathway that produces androgens and estrogens, Cytochrome P450, family 17, subfamily A, polypeptide 2 (Cyp17a2) possesses only 17α-hydroxylase activity and is known essential for the synthesis of cortisol. Besides with expressed in testes and ovaries, where the *cyp17a1* is mainly expressed, *cyp17a2* is also expressed in the interrenal gland in fish. Until now, the roles of *cyp17a2* in fish, especially in sexual traits development and hypothalamic-pituitary-interrenal (HPI) axis, are poorly studied. To investigate the roles of Cyp17a2 in teleosts, the *cyp17a2*-null zebrafish was generated and analyzed by us. The significantly decreased cortisol concentration was observed both in the *cyp17a2*-deficient males and females at adult stage. The interrenal gland enlargement, increased pituitary *proopiomelanocortin a* (*pomca*) expression, decreased locomotion activity and response to light-stimulated stress were observed in *cyp17a2*-deficient fish. Intriguingly, the *cyp17a2*-deficient males were fertile and with normal breeding tubercles on the pectoral fin, but females were infertile, deficient in genital papilla and with decreased gonadosomatic index (GSI). The increased progesterone (P4), 17α,20β-dihydroxy-4-pregnen-3-one (DHP) and 11-ketotestosterone (11-KT) in the *cyp17a2*-deficient males and females were observed. The increased concentration of testosterone (T) and estradiol (E2) was observed in *cyp17a2*-/- females and *cyp17a2*-/- males, respectively. By examining the ovaries development of *cyp17a2*-deficient fish at 3 months postfertilization (mpf), we observed that the oocytes were over-activated. Taken together, our findings demonstrate that Cyp17a2 is indispensable for production and physiology of cortisol, and *cyp17a2*-deficiency resulted in diminished cortisol but accumulated P4 and DHP, which may result in the over-activated oocytes in *cyp17a2*-deficient females.

## Introduction

The biosynthesis of gonadal and adrenal/interrenal hormones is basically conserved between mammals and fish. One of the featured distinctions is Cytochrome P450, family 17, subfamily A (Cyp17a). In human, Cyp17a1 catalyzes two major reactions: steroid 17-hydroxylation followed by the 17,20-lyase reaction in gonadal tissue (1). However, several fish have been reported to possess two Cyp17a paralogs: Cyp17a1 and Cyp17a2. It has been reported that Cyp17a1 has both 17α-hydroxylase and 17,20-lyase activities, which are important in the steroidogenic pathway that produces androgens. However, Cyp17a2 possesses only 17α-hydroxylase activity, and is required for the synthesis of cortisol. Besides with expressed in testes and ovaries, where the *cyp17a1* is mainly expressed, *cyp17a2* is also expressed in the interrenal gland and is probably important for the cortisol production in fish (2). Due to the discrepancies in the *cyp17a* gene profiles between mammals and teleosts, as well as the limited information about *cyp17a2* on zebrafish, it would be interesting to elucidate the underlying function of *cyp17a2* in the maintenance of hypothalamic-pituitary-interrenal (HPI) axis physiology and sexual traits development in fish.

Congenital adrenal hyperplasia (CAH) and diminished cortisol were seen in human beings with 17-hydroxylase/17,20-lyase deficiency (1). The majority of CAH is recorded to be caused by 21-hydroxylase (Cyp21a2) deficiency (21OHD), which is a group of common inherited disorders leading to glucocorticoid deficiency (3, 4). Cyp21a2 catalyzes the conversion of 17-hydroxyprogesterone to 11-deoxycortisol and progesterone to 11-deoxycorticosterone, which is cortisol and aldosterone precursor, respectively (5). In patients with 21OHD, the impaired synthesis of cortisol and overproduction of pituitary adrenocorticotropic hormone (ACTH), as well as overproduction of cortisol precursors with diverted biosynthesis of sex hormones were observed (4).

In teleost fish, cortisol is the main glucocorticoid produced in the interrenal gland (equivalent of the mammalian adrenal gland), and regulate a wide range of physiological effects, including body homeostasis and the response of the organism to external stressors (6). Zebrafish is an excellent model for the investigation of the catalyzing steps of the interrenal steroidogenic pathway of glucocorticoid biosynthesis. The production of cortisol in interrenal gland is regulated by hypothalamus and pituitary, formed the HPI axis (7–9). In *pomca*-deficient zebrafish, the decreased content of cortisol and increased testosterone, accompanied by impaired melanosome dispersal and reduced swim velocity were observed, suggesting the involvement of HPI axis in sex steroid production in zebrafish (9).

The Cytochrome P450 side-chain cleavage enzyme is reported to be crucial in interrenal and gonadal steroidogenesis, and *cytochrome P450, family 11, subfamily A, polypeptide 2* (*cyp11a2*)-deficiency resulted in decreased cortisol concentration, increased pituitary *pomca* expression, and impaired sex steroid deficiency and all-male fish with feminized secondary sex characters (10). It was reported that the knockout of *cytochrome P450, family 11, subfamily C, polypeptide 1* (*cyp11c1*), which encodes 11β-hydroxylase essential for the biosynthesis 11-KT and cortisol, caused interrenal gland enlargement in fish at 5 days postfertilization (dpf) and a reduction in egg spawning and a failure of *in vitro* germinal vesicle breakdown (GVBD) due to cortisol insufficiency at adult stage, which could be partially rescued by cortisol treatment (11). The interrenal gland enlargement is also observed in the *cyp21a2*-deficient zebrafish, which displayed several systemic hallmark features of human 21OHD (12).

In humans, it has been reported previously that the increased progesterone, 11-deoxycorticosterone and corticosterone, which accumulated upstream of the product catalyzed by Cyp17a1, can be seen due to the 17α-hydroxylase/17, 20-lyase deficiency caused by the *cyp17a1* mutation (1). The adrenal 17-hydroxylase activity is required for the maintenance of the glucocorticoid and mineralocorticoid homeostasis in human. The lack of adrenal 17α-hydroxylase activity (17α-hydroxylase deficiency, 17OHD) causes the production of corticosterone *via* 11-deoxycorticosterone rather than cortisol.

Though Cyp17a2 has been known as essential for steroidogenesis in gonad and interrenal gland of fish (2), the function of *cyp17a2* in fish with genetic manipulation method in HPI axis physiology and sexual traits development are unclear yet. In the present study, to investigate the function of Cyp17a2 in zebrafish, Clustered Regularly Interspaced Short Palindromic Repeats/CRISPR-associated system (CRISPR/Cas9) was used to knockout *cyp17a2*. The results provided solid evidence supporting that *cyp17a2* is indispensable in biosynthesis of cortisol, and its deficiency affects HPI axis physiology both in males and females, and hypothalamic-pituitary-gonad axis physiology in females.

## Materials and Methods

### Animals

Wild-type (WT) AB strain zebrafish (*Danio rerio*) were reared and utilized in the present study for the *cyp17a2* knockout. The zebrafish were maintained as previously described (13). Briefly, AB zebrafish were maintained under standard conditions at 28.5 °C. The fish were kept in a circulated water system with 14 hours light and 10 hours dark cycle. The *cyp17a2* heterozygous males and females in the F1 population were incrossed to generate an F2 population that contained *cyp17a2* homozygotes. All the experiments were performed in the fish of F2 population, which contained the fish with genotypes of *cyp17a2*+/+, *cyp17a2*+/- and *cyp17a2*-/-. The *cyp17a2*+/+ siblings at the corresponding stages with *cyp17a2*-/- fish served as control. All the fish experiments were conducted in accordance with the Guiding Principles for the Care and Use of Laboratory Animals and were approved by the Institute of Hydrobiology, Chinese Academy of Sciences (Approval ID: IHB 2013724).

### *cyp17a2* Knockout

The CRISPR/Cas9 strategy was utilized for the *cyp17a2* knockout. Guide RNA (gRNA), generated against the *cyp17a2* target sequence in the second exon, was designed using an online tool software (http://zifit.partners.org/ZiFiT) (14). The gRNA targeting site on the genomic DNA of the *cyp17a2* second exon adjacent to the Protospacer Adjacent Motif region (NGG) is as follows: GGGGCAGAGAGTTCGCCGGA. gRNA was synthesized from the polymerase chain reaction (PCR) products of the pMD19T-gRNA plasmid using the primers containing the target sequence and nominated sequence for the plasmid. PCR products were used for gRNA synthesis directly using the TranscriptAid T7 High Yield Transcription Kit following the manufacturer’s instructions (K0441, Thermo Fisher Scientific, Waltham, MA, USA). gRNA for the target site was transcribed and injected into embryos with Cas9 Nuclease and buffer (E365, Novoprotein, Suzhou, China). The Cas9 Nuclease, gRNA for *cyp17a2* and buffer were mixed for injection immediately before use. The final concentration of Cas9 and gRNA is 200 ng/μL and 100 ng/μL, respectively.

### Genotype Examination

For genotype examination, genomic DNA was used as the template for PCR, and the heteroduplex mobility assay (HMA) was performed, which is a rapid and sensitive analytical method for the detection of the genome modifications (15). Briefly, the polyacrylamide gel electrophoresis of the *cyp17a2* PCR products and imaging after ethidium bromide staining were used for genotype examination, as the heterologous chain in the *cyp17a2* PCR products of the heterozygote could be identified after the denaturation and renaturation. In contrast, a single band was observed in the PCR products of the *cyp17a2*+/+ fish and *cyp17a2*-/- fish. In the second round of PCR, the heterologous chain could be observed after PCR products of *cyp17a2*-/- fish were mixed with those of WT fish. The primers used for genotype examination of *cyp17a2* are listed in **Table 1**.

**Table 1:** Primers used in this study.

Gene	Primer direction^a^ and sequence (5’-3’)	Product (bp^b^)
**Genotyping**		
*cyp17a2*	F: GCAGCTGTCGTCTCAGTACG	149
	R: GGAGTGTGCTCACCATCTTG	
**qPCR**		
*pomca* (9)	F: TCTTGGCTCTGGCTGTTC	184
	R: TCGGAGGGAGGCTGTAG	
*lhβ* (9)	F: AGCTTGGTTTTTCCACGCTG	170
	R: TACGTGCACACTGTCTGGTG	
*fshβ* (9)	F: AGAGCGAAGAATGTGGGAGC	178
	R: GAATCAACCCCTGCAGGACA	
*gh1* (9)	F: GCATCAGCGTGCTCATCAAG R: TGAGACTGGTCTCCCCTACG	114
*prl* (9)	F: CTCAGCACCTCACTCACCAAT R: CAGAGACCGAGCCAATGACA	168
*tshβ* (9)	F: AGGTTGCCGTGCCTATGTG R: GGACCCACCAACTCCTTTATGT	145
*cyp17a1* (9)	F: CTCTTTGACCCAGGACGCTT R: TTTGCAAAATCCACGCCAGG	154
*β-actin* (9)	F: ACTCAGGATGCGGAAACTGG	119
	R: AGGGCAAAGTGGTAAACGCT	
*ef1α* (16)	F: AAGATCGGCTACAACCCTGC	107
	R: TTCCATCCCTTGAACCAGCC	
*gapdh* (17)	F: GATACACGGAGCACCAGGTT	158
	R: CAGGTCACATACACGGTTGC	

a F, Forward; R, Reverse.

b base pairs.

### Whole-Mount *in situ* Hybridization

We performed WISH as described previously (18). cDNAs of the following genes were used as antisense probes: *pomca*, *cyp11a1*, *cyp21a2* and *hydroxy-delta-5-steroid dehydrogenase, 3 beta- and steroid delta-isomerase 1* (*hsd3b1*). Firstly, the fixed larvae were washed with phosphate buffer with 0.1% Tween 20 (PBST), permeabilized with proteinase K, and hybridized with digoxigenin-labelled RNA probe at 65°C overnight. Secondly, after removal of the probe, larvae were incubated in hybridization buffer with gradually diminished saline sodium citrate (SSC) at 65°C, and in anti-digoxigenin-alkaline phosphatase, Fab fragments (11093274910, Roche, Basel, Switzerland) diluted in blocking solution overnight at 4°C. Thirdly, eight times wash with PBST followed by the NBT/BCIP Stock Solution (11681451001, Roche, Basel, Switzerland) diluted in alkaline phosphatase buffer was performed at room temperature. Finally, when the appropriate signal appeared, the reaction was terminated, and the images of the larvae were taken with 11.5× objective lenses of an Olympus microscope (Olympus, Tokyo, Japan). The pixel values of WISH signals were measured and quantified using National Institute of Health (NIH) ImageJ-analysis software.

### Whole-Body Sex Steroid Hormones Analyses

The concentrations of P4, T, 11-KT, E2, and cortisol from whole-body lysates of fish at 3 mpf were examined using commercial Enzyme Linked Immunosorbent Assay (ELISA) kits (P4: 582601, T: 582701, 11-KT: 582751, E2: 582251, and cortisol: 500360, Cayman Chemicals, Ann Arbor, MI, USA). The concentration of DHP from whole-body lysates of fish at 3 mpf was examined using commercial ELISA kits (498500, 498502 and 498504, Cayman Chemical Company). Briefly, the whole body of each fish was placed in a tube containing magnetic beads in a high-speed vortex destroyer instrument (Tissue Cell-destroyer 1000, Xinzongke viral Disease Control Bio-Tech LTD, Hubei, China) and homogenized in phosphate-buffer saline. After homogenization, an organic solvent was used to extract the sex steroids according to the manufacturer’s instructions. The layers were separated by vortexing and centrifugation, the organic layer was transferred to a fresh tube, and the extraction was repeated four times. The organic part was evaporated by heating to 30°C under a gentle stream of nitrogen. Finally, the extracts were dissolved in 200 µL ELISA buffer and prepared for measurement according to the manufacturer’s instructions.

### Locomotion Tracking Analysis

The locomotion was tracked and analyzed as described previously (9). Before the analysis, each fish at the adult stage was placed into a 20×10 cm tank and acclimatized for two hours. The locomotion activity was monitored using a ZebraTower system (ViewPoint Life Sciences, Montreal, Canada). The distance covered at large (> 5 cm/sec), slow-mild (2-5 cm/sec) and inactive (< 2 cm/sec) speed of *cyp17a2*+/+ fish and *cyp17a2*-/- fish were recorded for 5 minutes. Ten fish of each genotype were set as replicates.

### Fertility Assessment

The fertility was assessed by natural mating of the *cyp17a2*-deficient fish and WT fish in a spawning tank under standard aquarium conditions we previously described (19, 20).

### RNA Extraction and Quantitative Real-Time Polymerase Chain Reaction

Total RNA was isolated from the brains/pituitaries and ovaries of zebrafish by extraction with Trizol reagent (15596018, Ambion, TX, USA). A total of 500 ng of RNA template was used for reverse transcription and cDNA synthesis using a first-strand cDNA synthesis kit (K1622, Thermo Scientific, Massachusetts, USA). Each 20 μL amplification reaction contained 10 μL TransStart Tip Green qPCR SuperMix (AQ141, Transgen, Beijing, China), 0.5 μM of each forward and reverse primers and 2 μL of cDNA template. The qPCR was performed on a Bio-Rad real-time system (Bio-Rad Systems, Berkeley, CA, USA). All the mRNA levels were calculated as the fold expression relative to the housekeeping genes, *actin beta 1* (*β-actin*), *eukaryotic translation elongation factor 1 alpha 1* (*ef1a*) and *glyceraldehyde-3-phosphate dehydrogenase* (*gapdh*). The primers of *pomca*, *luteinizing hormone subunit beta* (*lhβ*), *follicle stimulating hormone subunit beta* (*fshβ*), *growth hormone 1* (*gh1*), *prolactin* (*prl*), *thyroid stimulating hormone subunit beta* (*tshβ*), *cyp17a1*, *β-actin*, *ef1α* and *gapdh* for qPCR are listed in **Table 1** (9, 16, 17).

### Hematoxylin and Eosin Staining

The dissected gonads were freshly fixed in 4% paraformaldehyde in phosphate-buffered saline at room temperature overnight followed by dehydration and infiltration. The staining procedure was performed as described in a previous study (21). Briefly, the samples were embedded and processed for paraffin sectioning using a microtome (Leica RM2235, Wetzlar, Germany). Paraffin sections of 5 μm in size were mounted on slides, deparaffinized, rehydrated, and washed with deionized water. The sections were stained with HE, dehydrated, mounted, and viewed under a Nikon ECLIPSE Ni-U microscope (Nikon, Tokyo, Japan). The scale bar has been provided in each image.

### Hydrocortisone Treatment

The *cyp17a2*-deficient females were treated with 20 nM hydrocortisone (20739, Cayman Chemicals, Ann Arbor, MI, USA) from 90 dpf to 100 dpf. Briefly, 10 fish were placed in a 3.5 L tank. Samples for genotyping were obtained using a tail fin cut, and the fertility capacity was recorded with mated with WT males.

The fish of F2 population were administrated with 100 nM hydrocortisone from 1 dpf to 5 dpf. WISH of fish at 5 dpf were then performed to assess the interrenal gland pattern of the hydrocortisone- and dimethyl sulfoxide (DMSO)-treated larvae.

### Statistical Analysis

The detailed information regarding the number of zebrafish used per experiment is provided in each experiment and the corresponding figure. All analyses were performed with the GraphPad Prism 6.0 software program and the differences were assessed using the Student’s *t*-test. The results were expressed as the mean ± SD. For all statistical comparisons, a *P* value < 0.05 was used to indicate a statistically significant difference.

## Results

### *cyp17a2* Knockout in Zebrafish

**Figure 1:**
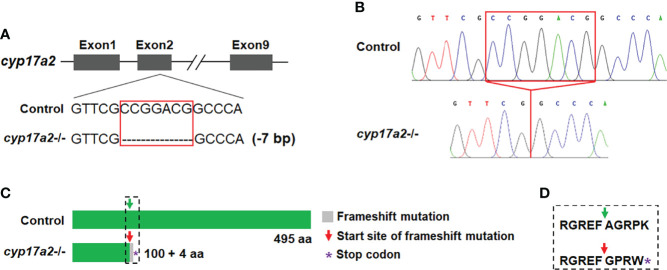
Deletion of *cyp17a2* in zebrafish by CRISPR/Cas9. **(A)** The gRNA target site of *cyp17a2* in the second exon. *cyp17a2*-null zebrafish with 7 base pairs deletion was obtained. **(B)** The representative chromatograms of the DNA sequences of control fish and *cyp17a2*-/- zebrafish, as evidenced by DNA sequencing of the PCR amplified DNA fragments using the genome DNA or cDNA as the template. The red squares in panel A and B indicate the 7 base pairs which have been deleted in *cyp17a2*-/- fish. **(C)** The predicted Cyp17a2 protein and mutant protein of *cyp17a2*-/- fish. The peptides of mutant Cyp17a2 identical to control Cyp17a2 protein are shown in green, the frameshift mutation is shown in gray. The premature stop codon is indicated with purple asterisk in panel **(C)** aa, amino acid. The amino acids before and after the start site of frameshift mutation in *cyp17a2*-/- fish and the corresponding area in the control fish is highlighted in dotted square and the detailed information is shown in panel **(D)**. **(D)** The detailed amino acid sequence of the dotted square in panel **(C)**.

*cyp17a2*, located on chromosome 23, encodes a protein of 495 amino acids. Utilizing the CRISPR/Cas9 strategy, the target knockout site harboring the second exon of *cyp17a2* was established by microinjection of recombinant protein of Cas9 nuclease and gRNA targeting *cyp17a2*. The F0 male fish with effective *cyp17a2* mutation at 3 mpf were crossed with WT females, and the offsprings were reared up and termed as F1 population. F2 fish with the genotypes of *cyp17a2*+/+, *cyp17a2*+/- and *cyp17a2*-/- were obtained from the crossing between the F1 heterozygotes at 3 mpf. The *cyp17a2*-null zebrafish with 7 base pairs (bp) deletion and frameshift mutation was obtained (**Figure 1A**). The PCR products amplified using the genome DNA or cDNA as the template were used for mutation validation (**Figure 1B**). The *cyp17a2*-null zebrafish only retained 100 correct amino acids, and after 4 incorrect amino acids, premature stopping occurred in *cyp17a2*-deficient fish (**Figures 1C, D**).

### The Interrenal Gland Enlargement Observed in *cyp17a2*-/- Fish At Larval Stage

**Figure 2:**
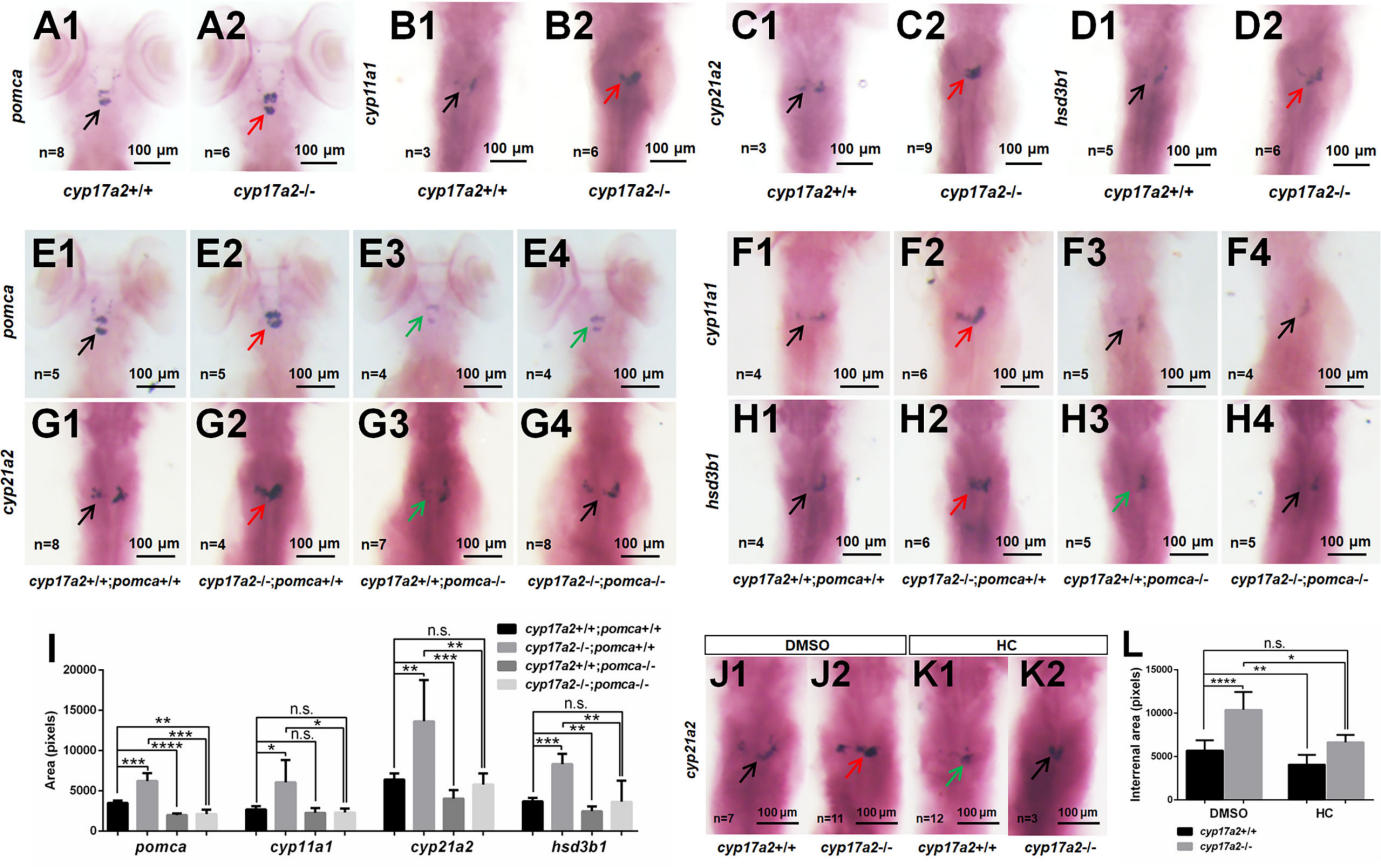
*cyp17a2*-deficiency caused pituitary *pomca* upregulation and interrenal gland enlargement in zebrafish larvae. WISH analyses with probes of *pomca* **(A1, A2)**, *cyp11a1* **(B1, B2)**, *cyp21a2* **(C1, C2)** and *hsd3b1* **(D1, D2)** in *cyp17a2*+/+ and *cyp17a2*-/- fish at 5 dpf. WISH analyses with probes of *pomca* **(E1–E4)**, *cyp11a1* **(F1–F4)**, *cyp21a2* **(G1–G4)**, and *hsd3b1* **(H1–H4)** in fish at 5 dpf. **(E1, F1, G1, H1)** *cyp17a2*+/+;*pomca*+/+ fish. **(E2, F2, G2, H2)** *cyp17a2*-/-;*pomca*+/+ fish. **(E3, F3, G3, H3)** *cyp17a2*+/+;*pomca*-/- fish. **(E4, F4, G4, H4)** *cyp17a2*-/-;*pomca*-/- fish. Black, red, and green arrows indicate normal, enlarged, and decreased interrenal gland, respectively. **(I)** Bar chart represents the quantification and statistical analyses of WISH signals in pituitary and interrenal gland of fish at 5 dpf. **(J1, J2, K1, K2)** WISH analysis using the probe of *cyp21a2* in *cyp17a2*+/+ and *cyp17a2*-/- fish administrated with DMSO or HC from 1 dpf to 5 dpf. **(J1, J2)** DMSO. **(K1, K2)** HC. **(L)** Bar chart represents the quantification and statistical analyses of WISH signals in interrenal gland of fish administrated with DMSO or HC. HC, hydrocortisone. DMSO, dimethyl sulfoxide. n.s., no significant difference. *P < 0.05. **P < 0.01. ***P < 0.001. ****P < 0.0001.

From WISH analyses using the probes of *pomca*, *cyp11a1*, *cyp21a2* and *hsd3b1*, we observed significantly upregulated expressions of pituitary *pomca* (**Figures 2A1, A2**) and interrenal gland *cyp11a1*, *cyp21a2* and *hsd3b1* (**Figures 2B1, B2, C1, C2, D1, D2**) in *cyp17a2*-deficient fish at 5 dpf. These results of *cyp17a2*-deficient fish at 5 dpf suggest that Cyp17a2 is important in interrenal gland development at larval stage, and the upregulated *pomca* expression may be caused by the steroidogenesis impairment, which resulted in the loss of negative feedback effect in the pituitary-interrenal axis.

Two independent *pomca* mutant lines with an 8 bp deletion and 8 bp insertion have been generated with transcription activator-like effector nucleases (TALENs) approach in our laboratory (9). In the present study, to answer whether upregulated *pomca* is responsible for the interrenal gland enlargement in the *cyp17a2*-deficient zebrafish, we introduced the *pomca* mutation of 8 bp deletion into the *cyp17a2*-deficient fish (9). To generate the *cyp17a2*-/-;*pomca*-/- fish, we incrossed the double heterozygotes (*cyp17a2*+/-;*pomca*+/-) among the F1 progeny. Among the F2 population, the *cyp17a2*-/-;*pomca*-/- fish could be obtained and used for the following analyses. From the statistical analysis of the WISH signals using the probes of *pomca*, *cyp11a1*, *cyp21a2* and *hsd3b1*, the upregulated pituitary *pomca* expression (**Figures 2E1, E2**) and interrenal gland *cyp11a1*, *cyp21a2* and *hsd3b1* expressions (**Figures 2F1, F2, G1, G2, H1, H2**) were observed in the *cyp17a2*-deficient fish. These genes, which were significantly upregulated in the *cyp17a2*-deficient fish at 5 dpf, could be effectively rescued by *pomca* knockout (in the *cyp17a2*-/-;*pomca*-/- fish) (**Figures 2E3, E4, F3, F4, G3, G4, H3, H4, I**).

Compared with that in *cyp17a2*-deficient fish reared in system water, the comparable expression level of interrenal gland *cyp21a2* was detected in control fish reared in system water with DMSO and *cyp17a2*-deficient fish reared in system water with hydrocortisone from 1 dpf to 5 dpf (**Figures 2J1, J2, K1, K2, L**). This result suggests that the interrenal gland enlargement in *cyp17a2*-deficient fish could be rescued by the hydrocortisone administration.

### The Impaired Sex Steroids Biosynthesis in *cyp17a2*-/- Fish at Adult Stage

**Figure 3:**
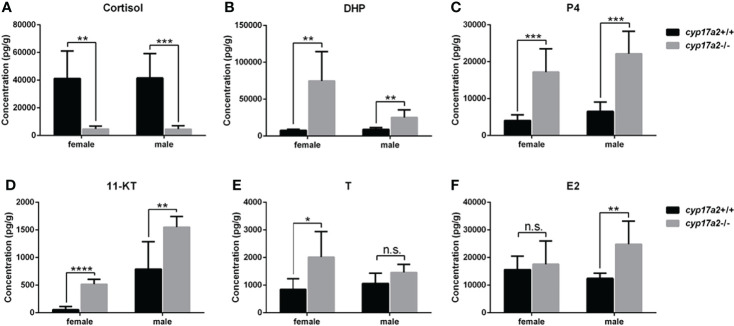
The steroid measurements. Whole-body levels of cortisol **(A)**, DHP **(B)**, P4 **(C)**, 11-KT **(D)**, T **(E)**, and E2 **(F)** in *cyp17a2*+/+ and *cyp17a2*-/- zebrafish at 3 mpf (n = 6). n.s., no significant difference. *P < 0.05. **P < 0.01. ***P < 0.001. ****P < 0.0001.

At adult stage, the body weight and body length between *cyp17a2*+/+ fish and *cyp17a2*-/- fish displayed no significant difference (**Supplemental Figure 1**). Subsequently, the whole-body cortisol, DHP, P4, 11-KT, T and E2 in the control fish and *cyp17a2*-/- fish at 3 mpf were measured using ELISA. Compared with their control siblings at their corresponding stages, a significant decrease in cortisol (**Figure 3A**), but increases in DHP, P4 and 11-KT were observed both in *cyp17a2*-/- males and females (**Figures 3B–D**). The increased T and E2 were observed in *cyp17a2*-/- females and males, respectively (**Figures 3E, F**). These results suggest that Cyp17a2 is indispensable in biosynthesis of steroids.

### The Impaired Locomotion Activity and Response to Light-Stimulated Stress in *cyp17a2*-/- Fish at Adult Stage

**Figure 4:**
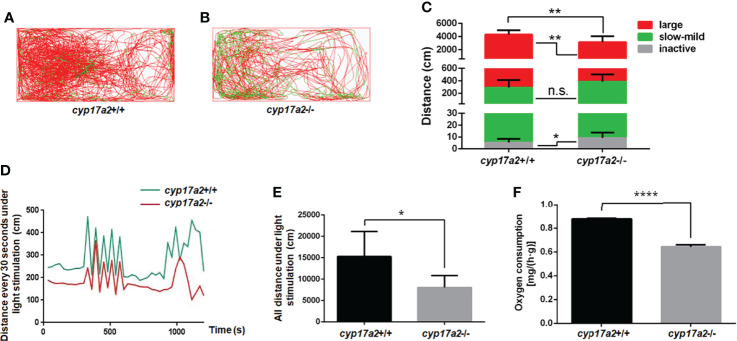
*cyp17a2*-deficiency resulted in impaired locomotion activity and stress response to light in zebrafish. **(A, B)** The locomotor trajectories of *cyp17a2*+/+ and *cyp17a2*-/- adult fish within 5 minutes (n = 10). **(C)** The distance fish covered at large, slow-mild, and inactive speed. The locomotor trajectories and distance covered at large (> 5 cm/sec), slow-mild (2-5 cm/sec) and inactive (< 2 cm/sec) speed in panel A-C are highlighted in red, green, and grey, respectively. **(D)** The movement distance curve analyzed in every 30 seconds under the light-stimulated stress within 20 minutes (n = 5). **(E)** All distance under the light-stimulated stress within 20 minutes (n = 5). **(F)** The oxygen consumption of *cyp17a2*+/+ and *cyp17a2*-/- fish after 2 days starvation (n = 3/group, 3 groups per genotype). n.s., no significant difference. *P < 0.05. **P < 0.01. ****P < 0.0001.

To confirm whether the other phenotypes related to cortisol-deficiency exists, we assessed the locomotion activity and reaction to cope with light-stimulated stress of the *cyp17a2*-/- fish. The total swimming distance and the distance covered at large speed were significantly lowered, but distance covered at inactive speed was significantly increased in the *cyp17a2*-/- fish (**Figures 4A–C**). The light-stimulated stress reaction in the *cyp17a2*-/- fish was also significantly downregulated, as evidenced with the distance statistically analyzed in every 30 seconds and in total (**Figures 4D, E**). Similar with *pomc*-deficient mice and zebrafish (9, 22), the decreased oxygen consumption was observed in the *cyp17a2*-/- fish (**Figure 4F**).

### Cyp17a2 Is Required for Oocyte Maturation and Secondary Sex Characters in Females

**Figure 5:**
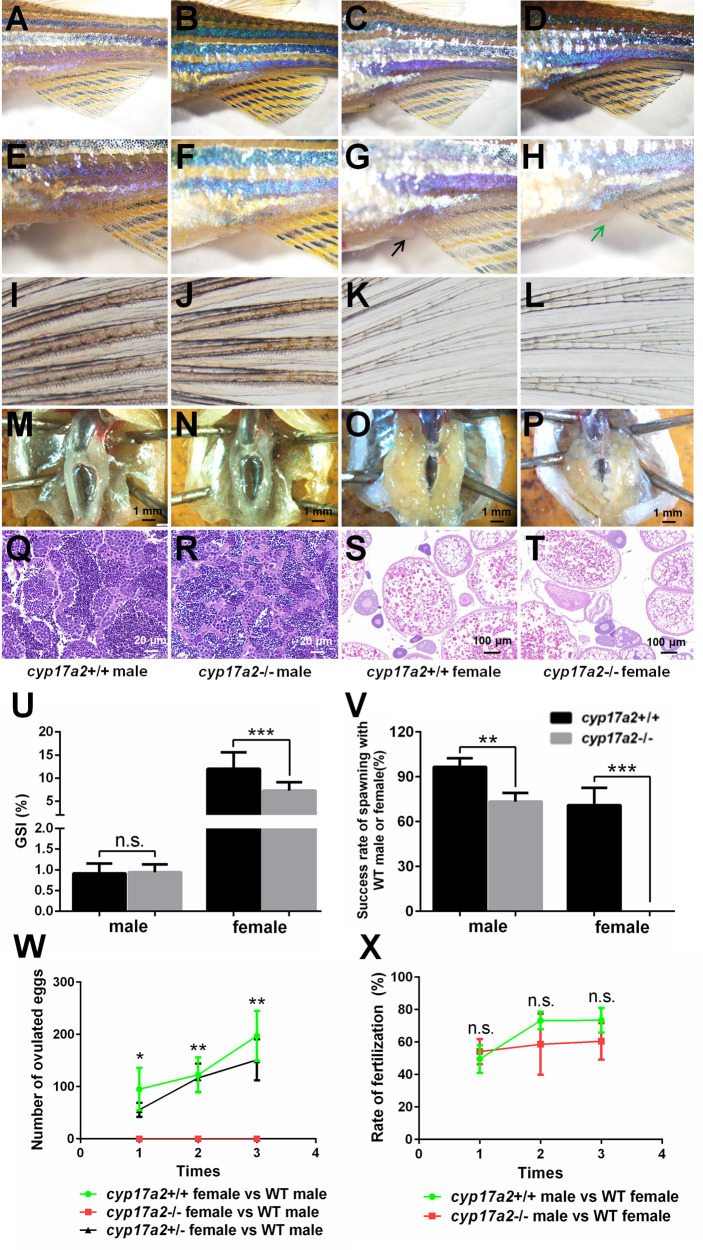
The analyses of primary and secondary sex characters and fertility assessment. **(A–D)** Gross appearance of *cyp17a2*+/+ male, *cyp17a2*-/- male, *cyp17a2*+/+ female, *cyp17a2*-/- female (n = 10). **(E–H)** The genital papilla. **(I–L)** The pectoral fin. **(M–P)** The anatomical observations. **(Q–T)** The histological analysis of gonads. Black and green arrow indicates normal and diminished genital papilla, respectively. **(U)** GSI of *cyp17a2*+/+ fish and *cyp17a2*-/- fish at 3 mpf (n > 10). **(V)** The fertility analysis of *cyp17a2*-/- males and *cyp17a2*-/- females mated with WT females and males, respectively (n = 10). **(W)** The statistical analyses of ovulated eggs of *cyp17a2*+/+, *cyp17a2*+/- and *cyp17a2*-/- females mated with WT males (n = 10). **(X)** The statistical analyses of fertilization ratio of the ovulated eggs from WT females when mated with *cyp17a2*+/+ males and *cyp17a2*-/- males, respectively (n = 10). n.s., no significant difference. *P < 0.05. **P < 0.01. ***P < 0.001.

The gross appearance of the control fish and *cyp17a2*-/- fish were examined at 3 mpf. The *cyp17a2*-deficient males presented no obvious difference with control males in male-typical secondary sex characters including normal breeding tubercles on the pectoral fin, and dark yellow coloration of the anal fin (**Figures 5A, B, E, F, I, J**). The *cyp17a2*-/- females showed no breeding tubercles on the pectoral fin and light-yellow coloration of the anal fin, but compromised genital papilla compared with control females (**Figures 5C, D, G, H, K, L**) and *cyp17a2*+/- females (data not shown). Both *cyp17a2*-/- males and females showed no obvious defects in gonads morphology as examined with anatomical examination (**Figures 5M–P**) and histological analysis (**Figures 5Q–T**). The *cyp17a2*-/- males and females displayed comparable and decreased GSI with control siblings, respectively (**Figure 5U**). When naturally mated with WT females and males, the spawning ratio of *cyp17a2*-/- males and females was decreased and diminished, respectively (**Figure 5V**). Compared with that in control females and *cyp17a2*+/- females, the number of ovulated eggs of *cyp17a2*-/- females was significantly and continuously diminished in the three periodically reproductive cycles when they were naturally mated with WT males (**Figure 5W**). In total, no progeny was obtained from the mating between the *cyp17a2*-/- females with WT males. The fertilization ratio of *cyp17a2*-/- males was comparable with control males when mated with WT females (**Figure 5X**).

**Figure 6:**
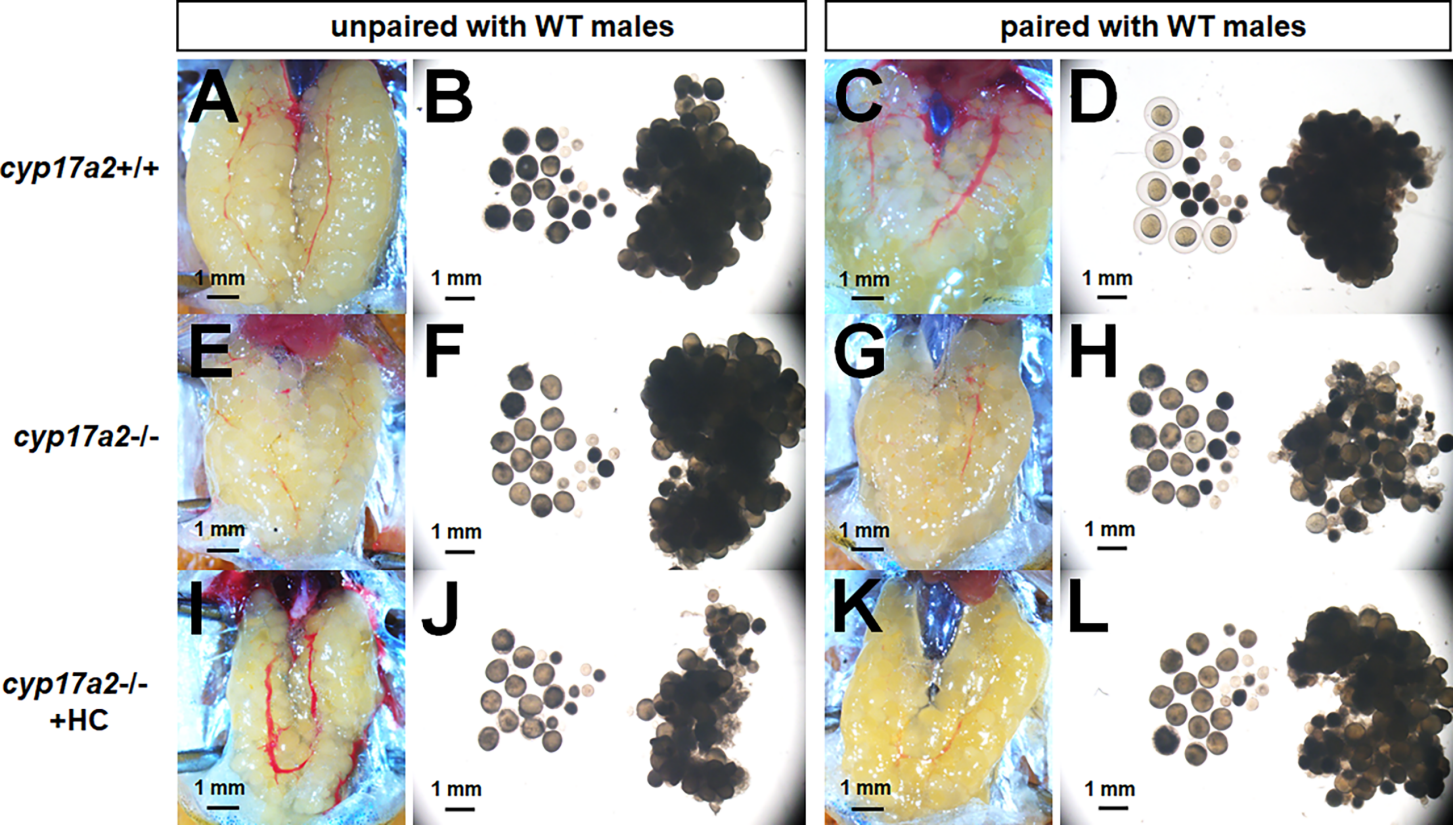
The over-activated oocytes in *cyp17a2*-/- females. **(A–D)** Under visual microscopy, the oocytes from control females unpaired and paired with WT males were not transparent and transparent, respectively (n = 5). **(E–H)** The oocytes from *cyp17a2*-/- females unpaired and paired with WT males were both transparent (n = 5). **(I–L)** The 20 nM hydrocortisone treatment in *cyp17a2*-/- females did not promotes oocyte maturation as observed under visual microscopy (n = 5).

To further address the reasons responsible for the infertility of the *cyp17a2*-/- females, we examined oocytes development of *cyp17a2*-deficient females at 3 mpf. Under visual microscopy, it could be observed that the dissected eggs from control females paired with WT males became transparent (**Figures 6A–D**), suggesting that the oocytes are mature eggs ready for ovulating and spawning when male fish were paired in the breeding tank. However, the dissected oocytes from *cyp17a2*-/- females unpaired with WT males were obviously transparent (**Figures 6E–H**). Even though, compared with that of the control females, the membrane of eggs could not be formed in oocytes from *cyp17a2*-/- females. These results suggest that the oocyte status in *cyp17a2*-deficient female zebrafish is aberrant: though oocytes have been over-activated in *cyp17a2*-deficient females when unpaired with WT males, their final maturation and fertilization are impaired. Furthermore, 20 nM hydrocortisone treatment in *cyp17a2*-/- females did not cause any obvious rescue effect in oocytes maturation under visual microscopy (**Figures 6I–L**) or ability in ovulation and spawning when mated with WT males (data not shown).

### The Compensatory Expression Profiles of Pituitary Hormones in *cyp17a2*-/- fish

**Figure 7:**
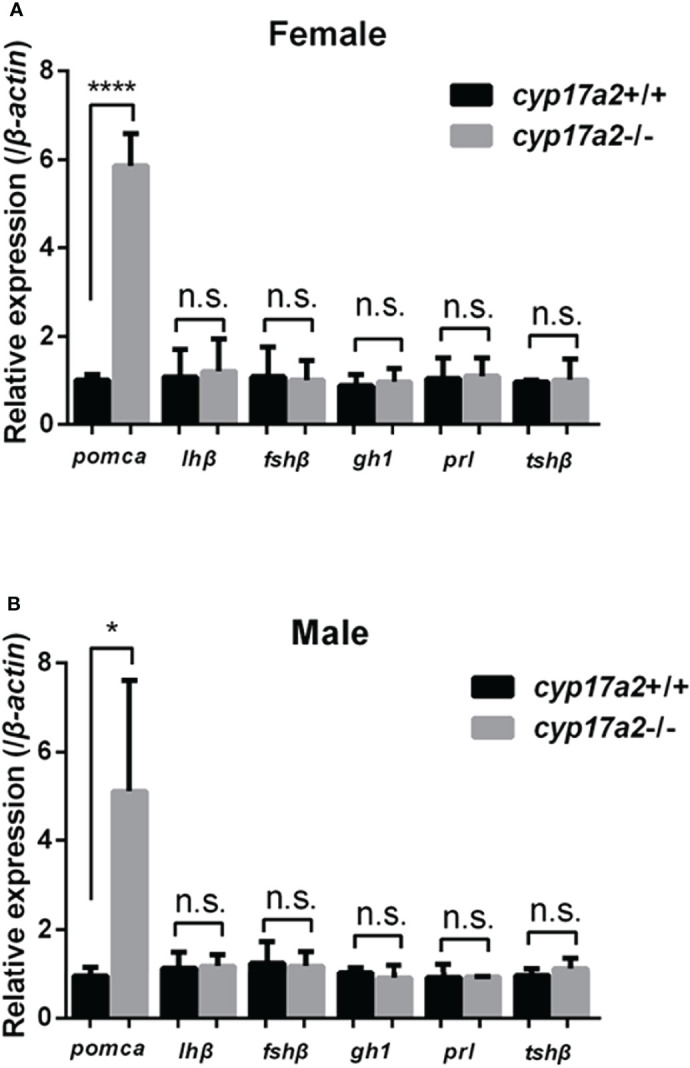
Expression profiles of pituitary hormones. Expressions of *pomca*, *lhβ*, *fshβ*, *gh1*, *prl* and *tshβ* in *cyp17a2*+/+ and *cyp17a2*-/- zebrafish at 3 mpf were analyzed with qPCR. All mRNA levels were calculated as the fold expression relative to the housekeeping gene *β-actin*. **(A)** Control females and *cyp17a2*-/- females. **(B)** Control males and *cyp17a2*-/- males. n.s., no significant difference. *P < 0.05. ****P < 0.0001.

The significantly diminished cortisol concentration and accumulated P4 and DHP in the *cyp17a2*-/- fish correlates with the phenotypes that were forementioned. To examine if compensatory expression in pituitary exists due to *cyp17a2* knockout-mediated steroids dysregulation, the expressions of *pomca*, *lhβ*, *fshβ*, *gh1*, *prl* and *tshβ* in pituitary of *cyp17a2*+/+ and *cyp17a2*-/- fish at 3 mpf were assessed. Compared with the expressions of these pituitary hormones in the control fish, only dramatically upregulated *pomca* in the pituitary of *cyp17a2*-deficient females and males was observed (**Figures 7A, B** and **Supplemental Figures 2A–D**).

## Discussion

Previously, we have characterized the role of Cyp17a1 in regulating sex differentiation, gonad development and secondary sex characters *via* catalyzing the androgens and estrogens in zebrafish and common carp (19, 23). Though several fish have been reported to possess two cyp17a paralogs: *cyp17a1* and *cyp17a2*, the function of *cyp17a2* in teleosts, which shed light on the underlying mechanism of the dual action of Cyp17a, especially the differentiated actions in the interrenal gland and gonad, has been little investigated. Here, we set out to uncover the role of *cyp17a2* in the aspects mentioned above and found that the phenotype of the interrenal gland enlargement and upregulated expression of pituitary *pomca* could be observed at larval stage (5 dpf). At adult stage, the decreased locomotion activity and light-stimulated stress reaction, as well as over-activated oocytes and compromised secondary sex characters in the *cyp17a2*-deficient females were observed.

Zebrafish possess two cyp17a paralogs, Cyp17a1 (possesses both 17α-hydroxylase and 17, 20-lyase activities) and Cyp17a2 (possesses only 17α-hydroxylase activity). Genome duplication occurred millions of years ago in the evolution of teleost fish may resulted in closely related paralogs involved in steroidogenesis. *cyp17a1* and *cyp17a2* are located on chromosome 13 and 23, respectively. Of the two genes expressed in gonads, *cyp17a2* is also exclusively expressed in interrenal gland, suggesting an indispensable role of *cyp17a2* in maintaining the biosynthesis of cortisol, the main circulating glucocorticoid in fish. Cyp17a2 catalyzes the conversion of pregnenolone and progesterone to 17OH-pregnenolone and 17OH-progesterone, the precursor of cortisol (2, 24–26). The *cyp17a2*-/- fish demonstrated a typical deficiency of cortisol biosynthesis (**Figure 3**). This could be supported by the other phenotypes related to cortisol deficiency, including decreased locomotion activity, response to light-stimulated stress and oxygen consumption in *cyp17a2*-/- fish (**Figure 4**). The decreased locomotion activity, response to light-stimulated stress and oxygen consumption observed in the *cyp17a2*-/- fish (**Figure 4F**) was similar with the observations of *pomc*-deficient zebrafish and mice (9, 22). These results suggest that the function of cortisol is conserved between mammals and teleosts.

Though Cyp17a2 has been identified in fish, it could not be obtained by searching in the National Center for Biotechnology Information (NCBI) database, as only steroid 17α-hydroxylase/17,20 lyase has been documented in the genomic database of several fish species, including *Cyprinus carpio*, *Ictalurus punctatus*, *Oncorhynchus mykiss*, etc. However, the phylogenetic tree analysis performed by us clearly revealed that they should be clustered into two orthologs, and one of them should be Cyp17a2, as referenced with the identified Cyp17a2 in zebrafish (*Danio rerio*) and tilapia (*Oreochromis niloticus*) (**Supplemental Figure 3**) (2, 24).

In human and other mammals, CAH represents inherited disorders that are characterized as impaired cortisol production by the adrenal gland (27), and occurs because of deficiency of the enzyme involved in the conversion of cholesterol to cortisol (28–30). In E5 of common carp (*Cyprinus carpio L*.), *P450c17a2* deficiency in expression resulted in high corticosterone and female to male sex reversal with interrenal hyperplasia (31). The enlargement of interrenal gland presented by *cyp17a2*-/- larvae at 5 dpf (**Figure 2**) correlates with the observations mentioned above (28–31). However, the sex reversal, which was seen in the E5 common carp, was not observed in the *cyp17a2*-deficient zebrafish, as they either developed into males or females. We think that the diverged phenotype of gonadal differentiation between *cyp17a2*-deficient zebrafish and E5 common carp may be owing to the different mutation locus, as the mutation responsible for *P450c17a2* expression is not clear yet, and Nematollahi et al. speculated that the mutation site may located in the promoter or control elements of *P450c17a2* (31).

Besides with *cyp17a2* mentioned above, several genes involved in steroidogenesis are expressed in interrenal gland at larval stage (5 dpf) and adult stage, including *steroidogenic acute regulatory protein* (*star*), *cyp11a1*, *cyp11a2*, *hsd3b1*, *cyp21a2*, and *cyp11c1* (5, 8, 11, 12, 32, 33). The TALENs, CRISPR/Cas9 or morpholino strategy was utilized for the knockout/knockdown of *star*, *cyp11a2*, *cyp21a2* and *hsd3b1*, and the mutants (or morphants) were found to be cortisol deficient with gonadal phenotype and compromised secondary sex characters (10, 12, 16, 34, 35). These results not only confirmed the role in steroidogenesis, but also demonstrated the role of steroids in gonadal development. Among these studies, deficient synthesis of cortisol, enlarged interrenal gland accompanied with increased pituitary *pomca* expression were observed in *hsd3b1*-deficient and *cyp21a2*-deficient zebrafish (11, 35). Previously, Weger et al. reported that the expression of *cyp17a2* could be detected in the interrenal gland at 5 dpf, suggesting an important role of *cyp17a2* in interrenal gland development or cortisol biosynthesis (33). The upregulated *pomca* expression in the pituitary of *cyp17a2*-deficient fish both at the larval stage and adult stage may be caused by the loss of negative feedback effect in the pituitary-internal axis and suggest that Cyp17a2 is important in the biosynthesis of cortisol in fish (**Figures 2**, **7**). The similar compensatory upregulation of pituitary gonadotropins, *lhβ* and *fshβ*, were observed in the *cyp17a1*-/- and *cyp19a1a*-/- fish, as well as upregulated pituitary hormone, *tshβ*, in *thyroglobulin*-/- and *monocarboxylate transporter 8*-/- fish. These suggest that the feedback mechanism exists in pituitary-gonad/interrenal/thyroid axis: when the effector organ is dysregulated in biosynthesis of sex steroids/corticosteroid/thyroxine, the pituitary hormones corresponding to the catalysate-deficiency would be upregulated due to feedback mechanism removal and usually accompanied with the enlargement of the gland (19, 36, 37).

Deleterious mutation or mutant mRNA degradation is known to cause genetic compensation of paralogues of genes (38, 39). We evaluated the expression of *cyp17a1* in the ovary of *cyp17a2*-deficient female; however, no compensative expression of *cyp17a1* was observed (**Supplemental Figure 4**). The reduced conversion of P4 to 17OH-progesterone by 17OHD is known to cause the accumulation of P4 and subsequent conversion by 21-hydroxylase into deoxy-corticosterone and corticosterone (1, 40). DHP is the major, potent, and biologically relevant progestin in teleosts (41–43), and the DHP content in organism with *cyp17a2*-deficiency has been poorly measured in previous studies. It has been known that in stage IV, Lhβ secreted from the pituitary, resume the meiotic cell cycle, and promotes GVBD, which is indicative of oocyte maturation (44). Previously, we have demonstrated that *star* is abundantly expressed in ovary, and *star*-deficiency or *lhβ*-deficiency mediated *star* downregulation both resulted in impaired oocyte maturation from stage IV due to insufficient sex steroids, P4, DHP or pregnenolone (P5). The effective restoration of the oocyte maturation defect could be observed with the administration of P4, DHP, and P5 on the *lhβ*- and *star*-deficient females (16).

After GVBD, the oocyte stepped into metaphase II, and the 1st polar body is extruded from the oocyte and the follicle expelled the oocyte (now properly termed an egg), which is fertilizable (44). In the present study, the increased concentration of progestins (P4 and DHP) correlates with the transparent oocytes of *cyp17a2*-/- female zebrafish (**Figures 3**, **6**). Interestingly, though the transparent oocytes with GVBD in *cyp17a2*-deficient females were seen, they were not fertilizable (data not shown). We think that the reasons attribute to the infertility of *cyp17a2*-/- females could be multiple, as both oocyte maturation and secondary sex characters were impaired. Notably, androgens (T and 11-KT) are accumulated in *cyp17a2*-/- females (**Figure 3**). We speculate that the endocrine or paracrine factor(s) relating to oocyte maturation may be impaired by the androgens accumulation or cortisol-insufficiency, which in turns resulted in the infertility of *cyp17a2*-deficient females. Of course, it is still necessary to analyze the oocyte maturation in *cyp17a2*-deficient females further, especially combine the analyses with *npgr*-/- or *lhβ*-/- female zebrafish.

Recently, utilizing the *cyp11c1*-and *cyp11a2*-null zebrafish, investigators demonstrated that the oocyte maturation was impaired due to insufficient biosynthesis of cortisol and P5, respectively (11, 45). To our knowledge, our *cyp17a2*-/- zebrafish is the first steroids-related gene knockout model that displayed over-activated oocytes due to accumulated progestins upstream of cortisol in fish. Therefore, we provided an excellent basis for mechanism investigation of the accumulated progestins in oocyte maturation of *cyp17a2*-/- female zebrafish.

In summary, based on the observations in the present study, it could be concluded that, compared with the male zebrafish, the females are more susceptible to *cyp17a2*-deficiency, as both ovarian development, female-typical secondary sex characters and fertility were impaired in the *cyp17a2*-/- females (**Figure 5**).

## Data Availability Statement

The original contributions presented in the study are included in the article/**Supplementary Material**. Further inquiries can be directed to the corresponding author.

## Ethics Statement

The animal study was reviewed and approved by the Institute of Hydrobiology, Chinese Academy of Sciences (Approval ID: IHB 2013724).

## Author Contributions

SS and TS conducted most of the experiments for this work. XL provided help in the analyses of fish movement and response to light-stimulated stress. QL, XJ and JH provided help in genotyping, fish breeding and rearing. ZY and GZ provided insights for this work, initiated and supervised the research team. GZ prepared all the figures and wrote the draft. ZY revised the paper. All authors approved the final manuscript and agreed to be accountable for all aspects of this work.

## Funding

This work was supported by the National Natural Science Foundation, China (No. 31972779), the Pilot Program A Project from the Chinese Academy of Sciences (No. XDA24010206), the National Key Research and Development Program, China (No. 2018YFD0900205), the Youth Innovation Promotion Association of CAS (2020336), and the State Key Laboratory of Freshwater Ecology and Biotechnology (2016FBZ05).

## Conflict of Interest

Author TS was employed by China Three Gorges Corporation.

The remaining authors declare that the research was conducted in the absence of any commercial or financial relationships that could be construed as a potential conflict of interest.

## Publisher’s Note

All claims expressed in this article are solely those of the authors and do not necessarily represent those of their affiliated organizations, or those of the publisher, the editors and the reviewers. Any product that may be evaluated in this article, or claim that may be made by its manufacturer, is not guaranteed or endorsed by the publisher.

## Supplementary Material

Supplementary Figure 1 The statistical analyses of body weight and body length. **(A)** Body weight of *cyp17a2*+/+ and *cyp17a2*-/- zebrafish at 3 mpf (n > 10). **(B)** Body length of *cyp17a2*+/+ and *cyp17a2*-/- zebrafish at 3 mpf (n > 10). n.s., no significant difference.

Supplementary Figure 2 Expressions of *pomca*, *lhβ*, *fshβ*, *gh1*, *prl* and *tshβ* were analyzed with qPCR. **(A, B)** All mRNA levels were calculated as the fold expression relative to the housekeeping gene *ef1α*. **(A)** Control females and *cyp17a2*-/- females. **(B)** Control males and *cyp17a2*-/- males. **(C, D)** All mRNA levels were calculated as the fold expression relative to the housekeeping gene *gapdh*. **(C)** Control females and *cyp17a2*-/- females. **(D)** Control males and *cyp17a2*-/- males. n.s., no significant difference. **P* < 0.05. *****P* < 0.0001.

Supplementary Figure 3 Phylogenetic analysis of amino acid sequences. The distance scale represents the degree of difference between sequences (*e.g.*, 0.10 indicates a 10% difference between two sequences). The gene definition and description have been provided after the name of the species. The defined Cyp17a1 or Cyp17a2 in previous publications have been highlighted in red.

Supplementary Figure 4 Expression of *cyp17a1* in ovary of *cyp17a2*+/+ females and *cyp17a2*-/- females at 3 mpf was analyzed with qPCR. The expression level of *cyp17a1* was calculated as the fold expression relative to the housekeeping gene *β-actin* **(A)**, *ef1α* **(B)**, and *gapdh* **(C)**, respectively. n.s., no significant difference.

## References

[1] Auchus RJ. Steroid 17-Hydroxylase and 17,20-Lyase Deficiencies, Genetic and Pharmacologic. J Steroid Biochem Mol Biol (2017) 165(Pt A):71–8. doi: 10.1016/j.jsbmb.2016.02.002 PMC4976049 26862015

[2] Zhou LY Wang DS Kobayashi T Yano A Paul-Prasanth B Suzuki A. A Novel Type of P450c17 Lacking the Lyase Activity is Responsible for C21-Steroid Biosynthesis in the Fish Ovary and Head Kidney. Endocrinology (2007) 148(9):4282–91. doi: 10.1210/en.2007-0487 17569754

[3] Krone N Arlt W. Genetics of Congenital Adrenal Hyperplasia. Best Pract Res Clin Endocrinol Metab (2009) 23(2):181–92. doi: 10.1016/j.beem.2008.10.014 PMC5576025 19500762

[4] Miller WL Auchus RJ. The Molecular Biology, Biochemistry, and Physiology of Human Steroidogenesis and its Disorders. Endocr Rev (2011) 32(1):81–151. doi: 10.1210/er.2010-0013 21051590 PMC3365799

[5] Bacila I Cunliffe VT Krone NP. Interrenal Development and Function in Zebrafish. Mol Cell Endocrinol (2021) 535:111372. doi: 10.1016/j.mce.2021.111372 34175410

[6] Dickmeis T. Glucocorticoids and the Circadian Clock. J Endocrinol (2009) 200(1):3–22. doi: 10.1677/JOE-08-0415 18971218

[7] Liu YW. Interrenal Organogenesis in the Zebrafish Model. Organogenesis (2007) 3(1):44–8. doi: 10.4161/org.3.1.3965 PMC2649615 19279699

[8] To TT Hahner S Nica G Rohr KB Hammerschmidt M Winkler C. Pituitary-Interrenal Interaction in Zebrafish Interrenal Organ Development. Mol Endocrinol (2007) 21(2):472–85. doi: 10.1210/me.2006-0216 17082325

[9] Shi C Lu Y Zhai G Huang J Shang G Lou Q. Hyperandrogenism in POMCa-Deficient Zebrafish Enhances Somatic Growth Without Increasing Adiposity. J Mol Cell Biol (2020) 12(4):291–304. doi: 10.1093/jmcb/mjz053 31237951 PMC7232124

[10] Li N Oakes JA Storbeck KH Cunliffe VT Krone NP. The P450 Side-Chain Cleavage Enzyme Cyp11a2 Facilitates Steroidogenesis in Zebrafish. J Endocrinol (2020) 244(2):309–21. doi: 10.1530/JOE-19-0384 31693487

[11] Zhang Q Ye D Wang H Wang Y Hu W Sun Y. Zebrafish Cyp11c1 Knockout Reveals the Roles of 11-Ketotestosterone and Cortisol in Sexual Development and Reproduction. Endocrinology (2020) 161(6):1–20. doi: 10.1210/endocr/bqaa048 32222764

[12] Eachus H Zaucker A Oakes JA Griffin A Weger M Guran T. Genetic Disruption of 21-Hydroxylase in Zebrafish Causes Interrenal Hyperplasia. Endocrinology (2017) 158(12):4165–73. doi: 10.1210/en.2017-00549 PMC5711382 28938470

[13] Westerfield M. The Zebrafish Book, a Guide for the Laboratory Use of Zebrafish (Danio Rerio). OR 4rd Ed. University of Oregon Press, Eugene (2000).

[14] Sander JD Zaback P Joung JK Voytas DF Dobbs D. Zinc Finger Targeter (ZiFiT): An Engineered Zinc Finger/Target Site Design Tool. Nucleic Acids Res (2007) 35(Web Server issue):W599–605. doi: 10.1093/nar/gkm349 PMC1933188 17526515

[15] Ota S Hisano Y Muraki M Hoshijima K Dahlem TJ Grunwald DJ. Efficient Identification of TALEN-Mediated Genome Modifications Using Heteroduplex Mobility Assays. Genes Cells (2013) 18(6):450–8. doi: 10.1111/gtc.12050 PMC4834911 23573916

[16] Shang G Peng X Ji C Zhai G Ruan Y Lou Q. Steroidogenic Acute Regulatory Protein and Luteinizing Hormone are Required for Normal Ovarian Steroidogenesis and Oocyte Maturation in Zebrafishdagger. Biol Reprod (2019) 101(4):760–70. doi: 10.1093/biolre/ioz132 31322169

[17] Liu L Zhu H Yan Y Lv P Wu W. Toxicity Evaluation and Biomarker Selection With Validated Reference Gene in Embryonic Zebrafish Exposed to Mitoxantrone. Int J Mol Sci (2018) 19(11):3516. doi: 10.3390/ijms19113516 PMC6274943 30413070

[18] Thisse C Thisse B. High-Resolution in Situ Hybridization to Whole-Mount Zebrafish Embryos. Nat Protoc (2008) 3(1):59–69. doi: 10.1038/nprot.2007.514 18193022

[19] Zhai G Shu T Xia Y Lu Y Shang G Jin X. Characterization of Sexual Trait Development in Cyp17a1-Deficient Zebrafish. Endocrinology (2018) 159(10):3549–62. doi: 10.1210/en.2018-00551 30202919

[20] Shu T Zhai G Pradhan A Olsson PE Yin Z. Zebrafish Cyp17a1 Knockout Reveals That Androgen-Mediated Signaling is Important for Male Brain Sex Differentiation. Gen Comp Endocrinol (2020) 295:113490. doi: 10.1016/j.ygcen.2020.113490 32283058

[21] Lau ES Zhang Z Qin M Ge W. Knockout of Zebrafish Ovarian Aromatase Gene (Cyp19a1a) by TALEN and CRISPR/Cas9 Leads to All-Male Offspring Due to Failed Ovarian Differentiation. Sci Rep (2016) 6:37357. doi: 10.1038/srep37357 27876832 PMC5120357

[22] Coll AP Challis BG Lopez M Piper S Yeo GS O'Rahilly S. Proopiomelanocortin-Deficient Mice are Hypersensitive to the Adverse Metabolic Effects of Glucocorticoids. Diabetes (2005) 54(8):2269–76. doi: 10.2337/diabetes.54.8.2269 16046291

[23] Zhai G Shu T Chen K Lou Q Jia J Huang J. Successful Production of an All-Female Common Carp (Cyprinus Carpio L.)population Using Cyp17a1-Deficient Neomale Carp. Engineering (2021) 8:181–9. doi: 10.1016/j.eng.2021.03.026

[24] Pallan PS Nagy LD Lei L Gonzalez E Kramlinger VM Azumaya CM. Structural and Kinetic Basis of Steroid 17alpha,20-Lyase Activity in Teleost Fish Cytochrome P450 17A1 and its Absence in Cytochrome P450 17a2. J Biol Chem (2015) 290(6):3248–68. doi: 10.1074/jbc.M114.627265 PMC4318999 25533464

[25] Ruane NM Th Goos HJ Komen J. Stress-Induced Facilitation of the Cortisol Response in 17alpha-Hydroxylase Deficient XX Mas-1/Mas-1 Carp (Cyprinus Carpio). Gen Comp Endocrinol (2007) 150(3):473–9. doi: 10.1016/j.ygcen.2006.11.008 17188688

[26] Nematollahi MA van Pelt-Heerschap H Komen J. Transcript Levels of Five Enzymes Involved in Cortisol Synthesis and Regulation During the Stress Response in Common Carp: Relationship With Cortisol. Gen Comp Endocrinol (2009) 164(1):85–90. doi: 10.1016/j.ygcen.2009.05.006 19463824

[27] Hall CM Jones JA Meyer-Bahlburg HF Dolezal C Coleman M Foster P. Behavioral and Physical Masculinization are Related to Genotype in Girls With Congenital Adrenal Hyperplasia. J Clin Endocrinol Metab (2004) 89(1):419–24. doi: 10.1210/jc.2003-030696 14715880

[28] New MI. Diagnosis and Management of Congenital Adrenal Hyperplasia. Annu Rev Med (1998) 49:311–28. doi: 10.1146/annurev.med.49.1.311 9509266

[29] Deaton MA Glorioso JE McLean DB. Congenital Adrenal Hyperplasia: Not Really a Zebra. Am Fam Physician (1999) 59(5):1190–6,1172. 10088875

[30] Yang X Iwamoto K Wang M Artwohl J Mason JI Pang S. Inherited Congenital Adrenal Hyperplasia in the Rabbit is Caused by a Deletion in the Gene Encoding Cytochrome P450 Cholesterol Side-Chain Cleavage Enzyme. Endocrinology (1993) 132(5):1977–82. doi: 10.1210/endo.132.5.7682938 7682938

[31] Nematollahi MA Pelt-Heerschap HV Komen H. High Corticosterone and Sex Reversal in Common Carp (Cyprinus Carpio L.) With Adrenal Hyperplasia Caused by P450c17a2 Deficiency. Aquaculture (2014) 418–419(1):165–70. doi: 10.1016/j.aquaculture.2013.10.011

[32] Bauer MP Bridgham JT Langenau DM Johnson AL Goetz FW. Conservation of Steroidogenic Acute Regulatory (StAR) Protein Structure and Expression in Vertebrates. Mol Cell Endocrinol (2000) 168(1-2):119–25. doi: 10.1016/S0303-7207(00)00316-6 11064158

[33] Weger M Diotel N Weger BD Beil T Zaucker A Eachus HL. Expression and Activity Profiling of the Steroidogenic Enzymes of Glucocorticoid Biosynthesis and the Fdx1 Co-Factors in Zebrafish. J Neuroendocrinol (2018) 30(4):e12586. doi: 10.1111/jne.12586 29486070

[34] Parajes S Griffin A Taylor AE Rose IT Miguel-Escalada I Hadzhiev Y. Redefining the Initiation and Maintenance of Zebrafish Interrenal Steroidogenesis by Characterizing the Key Enzyme Cyp11a2. Endocrinology (2013) 154(8):2702–11. doi: 10.1210/en.2013-1145 23671259

[35] Lin JC Hu S Ho PH Hsu HJ Postlethwait JH Chung BC. Two Zebrafish Hsd3b Genes Are Distinct in Function, Expression, and Evolution. Endocrinology (2015) 156(8):2854–62. doi: 10.1210/en.2014-1584 PMC4511139 25974401

[36] Tang H Chen Y Liu Y Yin Y Li G Guo Y. New Insights Into the Role of Estrogens in Male Fertility Based on Findings in Aromatase-Deficient Zebrafish. Endocrinology (2017) 158(9):3042–54. doi: 10.1210/en.2017-00156 28911176

[37] Song J Lu Y Cheng X Shi C Lou Q Jin X. Functions of the Thyroid-Stimulating Hormone on Key Developmental Features Revealed in a Series of Zebrafish Dyshormonogenesis Models. Cells (2021) 10(8):1984. doi: 10.3390/cells10081984 34440752 PMC8391828

[38] Rossi A Kontarakis Z Gerri C Nolte H Holper S Kruger M. Genetic Compensation Induced by Deleterious Mutations But Not Gene Knockdowns. Nature (2015) 524(7564):230–3. doi: 10.1038/nature14580 26168398

[39] El-Brolosy MA Kontarakis Z Rossi A Kuenne C Gunther S Fukuda N. Genetic Compensation Triggered by Mutant mRNA Degradation. Nature (2019) 568(7751):193–7. doi: 10.1038/s41586-019-1064-z PMC6707827 30944477

[40] Nematollahi MA van Pelt-Heerschap H Atsma W Komen J. High Levels of Corticosterone, and Gene Expression of Star, Cyp17a2, Hsd3b, Cyp21, Hsd11b2 During Acute Stress in Common Carp With Interrenal Hyperplasia. Gen Comp Endocrinol (2012) 176(2):252–8. doi: 10.1016/j.ygcen.2012.01.023 22333211

[41] Wang C Liu D Chen W Ge W Hong W Zhu Y. Progestin Increases the Expression of Gonadotropins in Pituitaries of Male Zebrafish. J Endocrinol (2016) 230(1):143–56. doi: 10.1530/JOE-16-0073 PMC4938713 27113852

[42] Scott AP Sumpter JP Stacey N. The Role of the Maturation-Inducing Steroid, 17,20beta-Dihydroxypregn-4-En-3-One, in Male Fishes: A Review. J Fish Biol (2010) 76(1):183–224. doi: 10.1111/j.1095-8649.2009.02483.x 20738705

[43] Chen SX Bogerd J Schoonen NE Martijn J de Waal PP Schulz RW. A Progestin (17alpha,20beta-Dihydroxy-4-Pregnen-3-One) Stimulates Early Stages of Spermatogenesis in Zebrafish. Gen Comp Endocrinol (2013) 185:1–9. doi: 10.1016/j.ygcen.2013.01.005 23360837

[44] Clelland E Peng C. Endocrine/paracrine Control of Zebrafish Ovarian Development. Mol Cell Endocrinol (2009) 312(1-2):42–52. doi: 10.1016/j.mce.2009.04.009 19406202

[45] Wang Y Ye D Zhang F Zhang R Zhu J Wang H. Cyp11a2 Is Essential for Oocyte Development and Spermatogonial Stem Cell Differentiation in Zebrafish. Endocrinology (2022) 163(2):1–17. doi: 10.1210/endocr/bqab258 34932120

