# Supplementary figures and images for: Characterization of the Interrenal Gland and Sexual Traits Development in cyp17a2-Deficient Zebrafish
Source: Front Endocrinol (Lausanne). 2022 Jun 6;13:910639. doi: 10.3389/fendo.2022.910639 (PMC9207535; doi:10.3389/fendo.2022.910639)

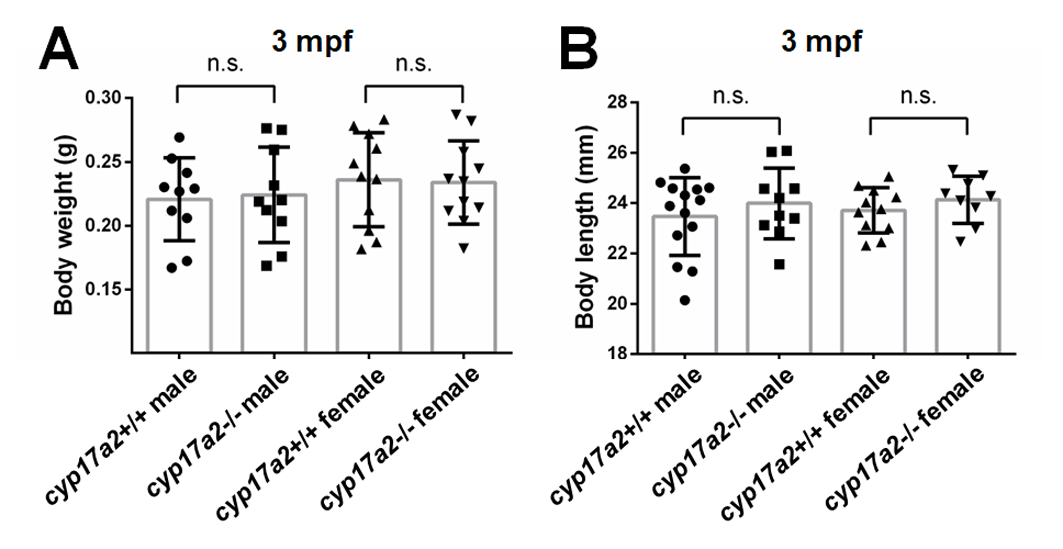

Supplement: Supplementary Figure 1 — The statistical analyses of body weight and body length. (A) Body weight of cyp17a2+/+ and cyp17a2-/- zebrafish at 3 mpf (n > 10). (B) Body length of cyp17a2+/+ and cyp17a2-/- zebrafish at 3 mpf (n > 10). n.s., no significant difference. [file Image_1.tif]

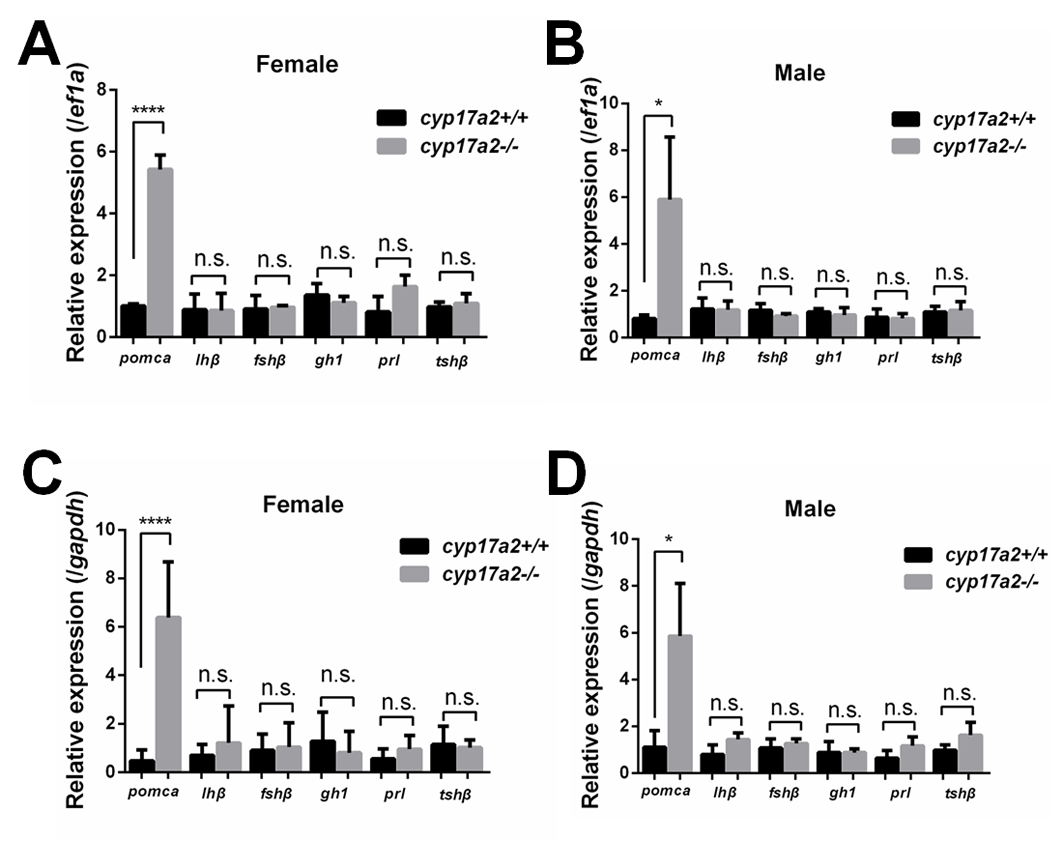

Supplement: Supplementary Figure 2 — Expressions of pomca, lhβ, fshβ, gh1, prl and tshβ were analyzed with qPCR. (A, B) All mRNA levels were calculated as the fold expression relative to the housekeeping gene ef1α. (A) Control females and cyp17a2-/- females. (B) Control males and cyp17a2-/- males. (C, D) All mRNA levels were calculated as the fold expression relative to the housekeeping gene gapdh. (C) Control females and cyp17a2-/- females. (D) Control males and cyp17a2-/- males. n.s., no significant difference. *P < 0.05. ****P < 0.0001. [file Image_2.tif]

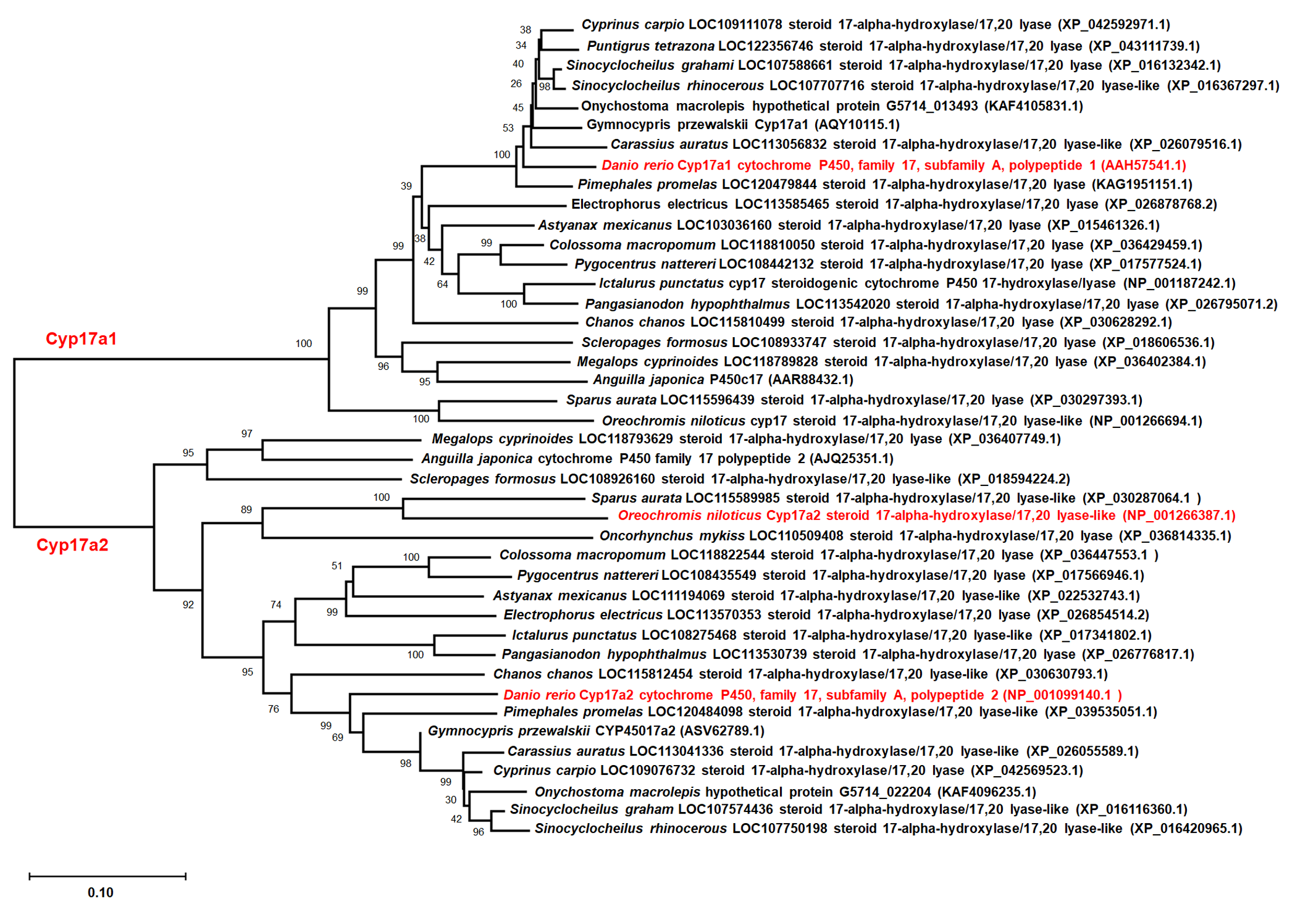

Supplement: Supplementary Figure 3 — Phylogenetic analysis of amino acid sequences. The distance scale represents the degree of difference between sequences (e.g., 0.10 indicates a 10% difference between two sequences). The gene definition and description have been provided after the name of the species. The defined Cyp17a1 or Cyp17a2 in previous publications have been highlighted in red. [file Image_3.tif]

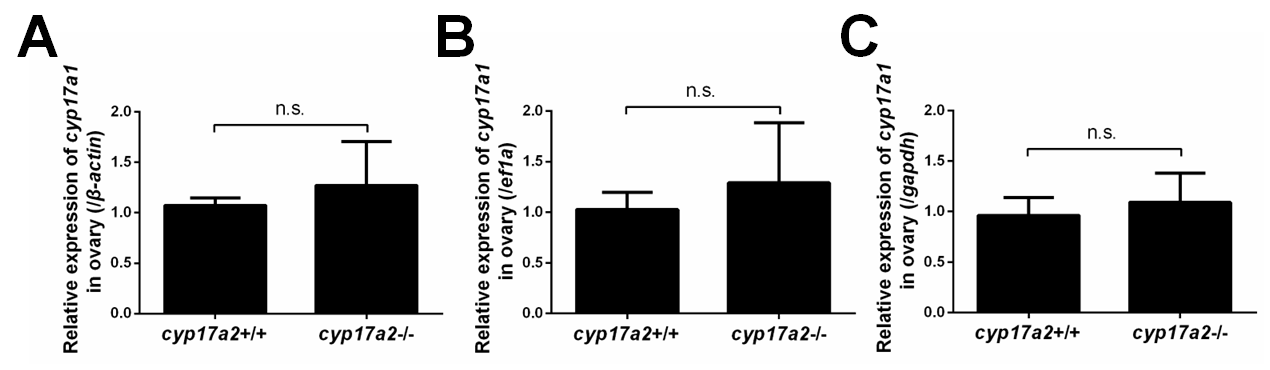

Supplement: Supplementary Figure 4 — Expression of cyp17a1 in ovary of cyp17a2+/+ females and cyp17a2-/- females at 3 mpf was analyzed with qPCR. The expression level of cyp17a1 was calculated as the fold expression relative to the housekeeping gene β-actin (A), ef1α (B), and gapdh (C), respectively. n.s., no significant difference. [file Image_4.tif]
